# Graphene as a Schottky Barrier Contact to AlGaN/GaN Heterostructures

**DOI:** 10.3390/ma13184140

**Published:** 2020-09-17

**Authors:** Maksym Dub, Pavlo Sai, Aleksandra Przewłoka, Aleksandra Krajewska, Maciej Sakowicz, Paweł Prystawko, Jacek Kacperski, Iwona Pasternak, Grzegorz Cywiński, Dmytro But, Wojciech Knap, Sergey Rumyantsev

**Affiliations:** 1 CENTERA Laboratories, Institute of High Pressure Physics PAS, ul. Sokołowska 29/37, 01-142 Warsaw, Poland; psai@unipress.waw.pl (P.S.); aleksandra.przewloka@gmail.com (A.P.); aleksandrababis@gmail.com (A.K.); gc@unipress.waw.pl (G.C.); but.dmitry@gmail.com (D.B.); knap.wojciech@gmail.com (W.K.); roumis4@gmail.com (S.R.); 2 V. E. Lashkaryov Institute of Semiconductor Physics, National Academy of Sciences of Ukraine, 41 pr. Nauki, 03680 Kyiv, Ukraine; 3 CEZAMAT, Warsaw University of Technology, 02-822 Warsaw, Poland; 4 Institute of Optoelectronics, Military University of Technology, gen. Sylwestra Kaliskiego 2 Str., 00-908 Warsaw, Poland; 5 Institute of High Pressure Physics PAS, ul. Sokołowska 29/37, 01-142 Warsaw, Poland; sakowicz400@gmail.com (M.S.); pprysta@unipress.waw.pl (P.P.); j.kacperski@topganlasers.com (J.K.); 6 Faculty of Physics, Warsaw University of Technology, 00-662 Warsaw, Poland; Iwona.Pasternak@pw.edu.pl; 7 Laboratoire Charles Coulomb, University of Montpellier and CNRS UMR 5221, 34950 Montpellier, France

**Keywords:** graphene, AlGaN/GaN, Schottky diode, field-effect transistor, finFET, noise

## Abstract

Electrical and noise properties of graphene contacts to AlGaN/GaN heterostructures were studied experimentally. It was found that graphene on AlGaN forms a high-quality Schottky barrier with the barrier height dependent on the bias. The apparent barrier heights for this kind of Schottky diode were found to be relatively high, varying within the range of φ_b_ = (1.0–1.26) eV. AlGaN/GaN fin-shaped field-effect transistors (finFETs) with a graphene gate were fabricated and studied. These devices demonstrated ~8 order of magnitude on/off ratio, subthreshold slope of ~1.3, and low subthreshold current in the sub-picoamperes range. The effective trap density responsible for the 1/f low-frequency noise was found within the range of (1–5) · 10^19^ eV^−1^ cm^−3^. These values are of the same order of magnitude as reported earlier and in AlGaN/GaN transistors with Ni/Au Schottky gate studied as a reference in the current study. A good quality of graphene/AlGaN Schottky barrier diodes and AlGaN/GaN transistors opens the way for transparent GaN-based electronics and GaN-based devices exploring vertical electron transport in graphene.

## 1. Introduction

The invention of graphene opened the road to new exciting physics and new devices based on the unique ultimate geometry of graphene and its specific physical properties and effects. Among them are extremely high carrier mobility [1,2,3], quantum Hall effect at room temperature [4], huge thermal conductivity [5], extreme sensitivity, and possible selectivity to ambient gases [6,7].

The majority of the graphene research deals with in-plane (lateral) current transport. A much less explored field is vertical transport through graphene and other two-dimensional materials. Indeed, there are just a few fields of research activity in this direction: transparent electrodes for light-emitting diodes and displays [8], vertical graphene-based hot electron transistors [9,10,11], and phase transitions in 2D transition metal chalcogenides [12].

Another promising field, which is the topic of the current letter, is graphene electrodes for electronic devices. It is known that graphene can form either an ohmic or Schottky barrier contact to semiconductors (see Reference [13] and references therein). This gives an opportunity to fabricate all transparent electrodes field-effect transistors and other devices. In the case of wide bandgap semiconductors, like GaN and SiC, which are transparent in the visible spectral range, it provides an opportunity for all transparent circuits. Graphene contacts withstand very high temperature [14], therefore graphene contact circuits are promising for high-temperature applications as well. 

The combination of graphene on the top of the AlGaN layer in AlGaN/GaN high-electron-mobility transistors (HEMTs) and 2D electron gas (2DEG) on AlGaN/GaN interface create a unique system with two closely spaced high-conductivity 2D layers. This kind of system is a promising platform to study the Coulomb interactions [15] and two-stream instability in the terahertz frequency band [16,17] in the system of massless electrons in graphene and 2D electrons in GaN. 

Although graphene/AlGaN/GaN heterostructures are promising for several applications, the properties of them are only preliminary studied.

In this work, we studied the properties of the graphene for AlGaN contact and the behavior of AlGaN/GaN finFETs with the graphene Schottky barrier gate. Special attention was paid to the low-frequency noise properties, which characterize the quality of the structures and are crucial for the majority of applications.

## 2. Device Fabrication and Experimental Details

**Figure 1:**
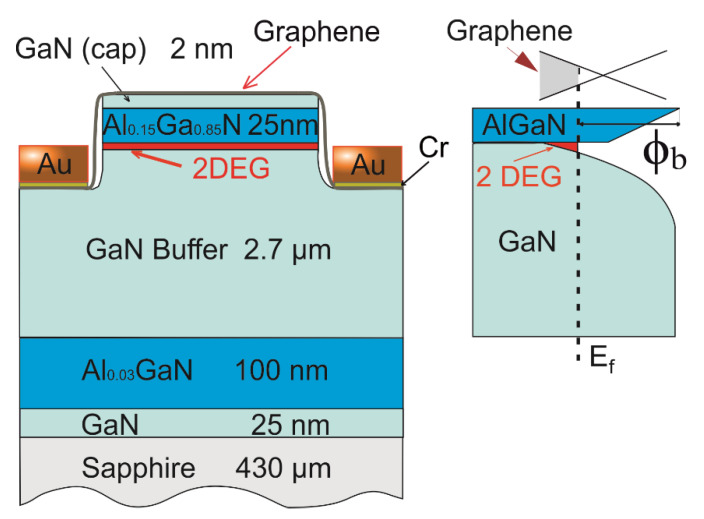
Layer structure and qualitative band diagram of graphene/AlGaN/GaN heterostructure used to study graphene/AlGaN Schottky barrier and graphene gate finFETs (not in scale).

The AlGaN/GaN structures were grown by the Metalorganic Vapour Phase Epitaxy method in the closed coupled showerhead 3 · 2 inch Aixtron reactor (Aixtron, Herzogenrath, Germany). The epistructure consisted of 2 nm GaN cap, 25 nm Al_0.15_Ga_0.85_N barrier layer, 2.7 μm intentionally undoped GaN layers, and 100 nm Al_0.03_Ga_0.97_N buffer. The schematic of the layer sequence and qualitative band diagram of the structure are shown in Figure 1. All epilayers were grown on the c-plane sapphire substrate.

The device processing was performed using a commercial laser writer system (Microtech, Palermo, Italy) for lithography processing with the 405 nm GaN laser source. In order to separate devices on the wafer, the shallow 150 nm mesas were etched using Inductively Coupled Plasma-Reactive Ion Etching (ICP-RIE) (Oxford Instruments, Bristol, UK) (also shown in Figure 1). The mesas had the shape of the rectangles with sides sized from 2 to 20 µm. Graphene layers were deposited first on the whole wafer and then patterned using oxygen plasma etching.

**Figure 2:**
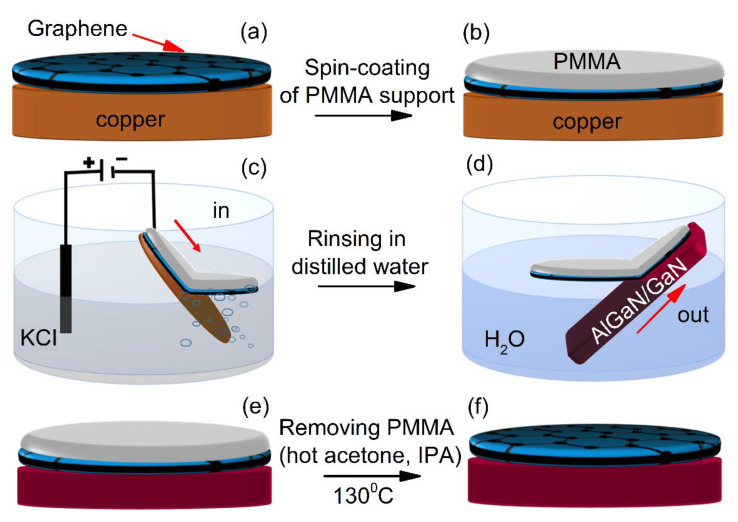
Main technological steps of graphene transfer procedure from copper foil to AlGaN/GaN structure. (**a**) graphene grown on copper; (**b**) PMMA coating; (**c**) Gr/PMMA is separated from Cu foil; (**d**) Gr/PMMA is transferred onto AlGaN/GaN structure; (**e**) Gr/PMMA onto AlGaN/GaN structure; (**f**) clean Gr/AlGa/GaN heterostructure.

We used a commercially available single-layer graphene (Graphenea, San Sebastián, Spain) produced by chemical vapor deposition (CVD) method on the surface of thin copper foils [18]. The step-by-step procedure of graphene transfer from a copper foil to AlGaN/GaN structures is shown in Figure 2. In order to detach graphene from Cu substrate, the high-speed electrochemical delamination (ED) process was used [19]. For this purpose, a thin layer of poly (methyl methacrylate) PMMA solution (4% in anisole) (MicroChemicals, Ulm, Germany) was spin-coated onto graphene/Cu stack. Then, using a custom-made mechanism, the Cu/Gr/PMMA stack was slowly and vertically immersed in potassium chloride solution (KCl, 1 mol/dm^3^) (Chempur, Piekary Śląskie, Poland) starting from the edge of the stack. As a result, Gr/PMMA separated from Cu foil and remained floating on the electrolyte surface. After that, the detached Gr/PMMA stack was moved to deionized water to rinse the electrolyte residue, transferred onto AlGaN/GaN structure, and heated at ~130 °C to make better contact. Finally, the PMMA support was carefully removed in acetone, and the structure with graphene was rinsed in IPA. This procedure allowed us graphene deposition on large area substrates of 1 inch or more in diameter. Measurements of the back-gate transistors fabricated using this graphene indicated its p-type. 

**Figure 3:**
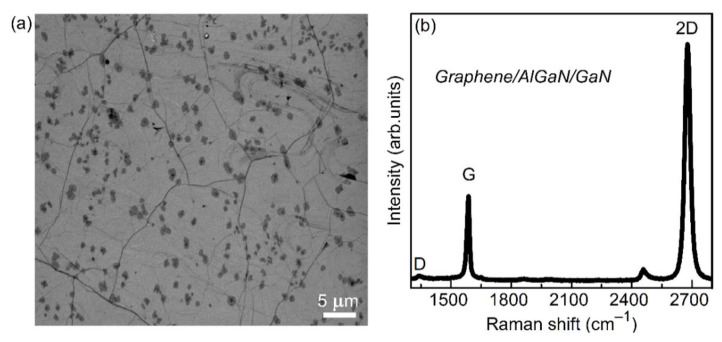
SEM image of graphene on the top of AlGaN/GaN structure (dark spots correspond to multilayer graphene islands) (**a**), Raman spectra of graphene (**b**).

The quality of the graphene layers was controlled with Raman spectroscopy and Scanning Electron Microscopy (SEM). The surface morphology of the transferred graphene samples was investigated by Auriga CrossBeam Workstation (Carl Zeiss) (Oberkoche, Germany). Figure 3a shows SEM images of graphene layers transferred onto AlGaN/GaN structure. Dark spots are regions of multilayer graphene, which are characteristic of graphene grown on copper foil. Raman spectra were measured with Renishaw InVia micro-Raman (Renishaw, Wotton-under-Edge, UK) using the 532 nm wavelength laser. The representative Raman spectrum of the graphene structure shown in Figure 3b includes three main peaks: G (1585 cm^−1^), 2D (2680 cm^−1^), and D (1350 cm^−1^). Low Full Width at Half Maximum (FWHM about 32 cm^−1^) of the 2D peak, as well as a much higher intensity of the 2D mode by comparison with the intensity of the G peak (2D/G > 2) are characteristic of the monolayer of graphene. Very low D mode amplitude indicates the high quality of the graphene structures. After high-speed electrochemical delamination, graphene was patterned in oxygen plasma using an Oxford Instruments Plasmalab 80 Plus ICP-RIE system (Oxford Instruments, Bristol, UK).

As a result of the etching, graphene remained on the mesas leaving the short ends of the rectangular mesas free from it. These parts of the mesas were used to fabricate ohmic contacts to 2DEG. In order to form ohmic contacts, Ti/Al/Ni/Au (150/1000/400/500 Å) layers were deposited and rapid thermal annealed at 800 °C in the nitrogen atmosphere for 60 s. 

Parts of the graphene remained out of the mesas on GaN buffer and were used to fabricate Cr/Au (50/1850 Å) ohmic contacts to it. The resulting design represents finFET with the graphene gate. This design allowed us to study the properties of the graphene AlGaN Schottky barrier and the characteristics of the finFETs themselves.

**Figure 4:**
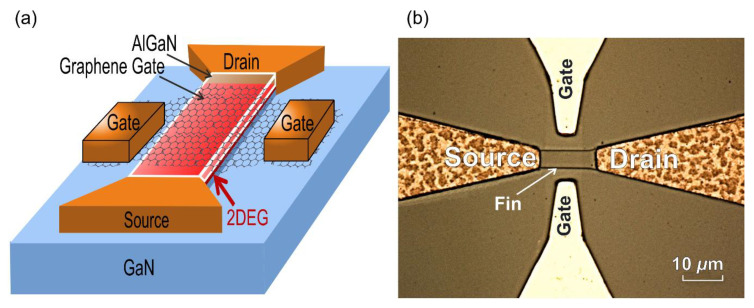
Schematic view (not in scale) (**a**) and optical microscope image (**b**) of the fabricated finFET with graphene gate.

The schematic view of the fabricated finFET and optical microscope image of one of the graphene gate transistors are shown in Figure 4a,b, respectively. The graphene gate is seen in Figure 4b as a low contrast rectangular over the fin. The gate width and length of the fabricated transistors were L_g_ = 5–20 µm and W_g_ = 4–20 µm, respectively. As a reference, finFETs with standard Ni/Au (250/750 Å) Schottky barrier gates were also fabricated.

All measurements were done at room temperature using a probe station. The spectral noise density of drain current fluctuations was calculated as $S_{I}=S_{v}{\left[\frac{R_{L\;}+{\;R}_{d}}{R_{L}R_{d}}\right]}^{2}$, where S_v_ is the drain voltage fluctuations and R_d_ is the differential device resistance.

## 3. Results and Discussion

**Figure 5:**
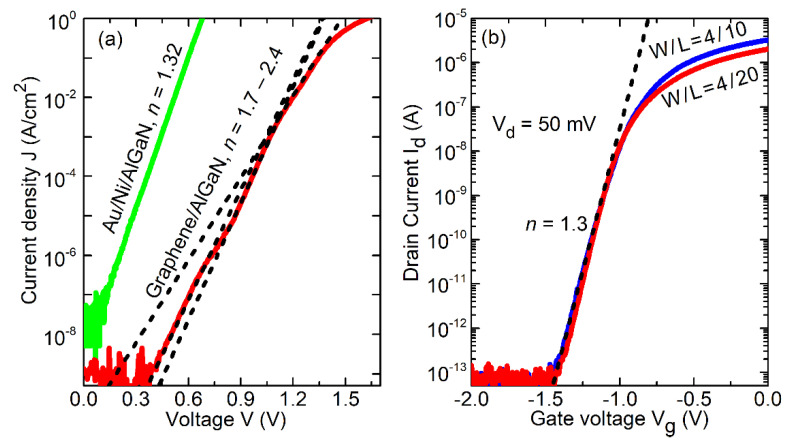
Current–voltage characteristics of Schottky diodes with Ni/Au contact (green) and graphene contact (red) lines (**a**). Transfer current–voltage characteristics of finFETs with graphene gate (**b**).

Figure 5a shows examples of the current–voltage characteristics of graphene/AlGaN and Au/Ni/AlGaN Schottky barrier diodes.

As seen, the Au/Ni/AlGaN Schottky barrier diode demonstrates exponential forward current–voltage characteristics I–exp (V_g_). In accordance with the thermionic emission (TE) model, the current–voltage characteristic of the Schottky diode at $V_{g}>k_{B}\cdot T/q$ is given by:

(1) $$I=I_{0}\cdot \exp\left(\frac{q\cdot V}{n\cdot k_{B}\cdot T}\right)$$

where $I_{0}=A^{*\;}T^{2}\cdot S\cdot \exp\left(-\frac{q\cdot \;\phi_{b}}{k_{B}\cdot \;T}\right)$ is the saturation current, q is the elemental charge, V is the applied voltage, n is the ideality factor, k_B_ is the Boltzmann constant, T is the temperature, A* is the modified Richardson constant, S is the contact area, and φ_b_ is the barrier height. Although under the TE model the ideality factor n = 1, experimental characteristics are always characterized by n > 1. The higher than unity ideality factor can be attributed to the effects of image force barrier lowering and the non-idealities of the barrier, such as inhomogeneity of the barrier height and the contribution of other current mechanisms (trap assisted tunneling, for example). Therefore, the ideality factor is often used as a figure of merit of the barrier quality. The current-voltage characteristic of Au/Ni/AlGaN Schottky barrier diode in Figure 5a demonstrates an ideality factor of n = 1.32. The whole range of ideality factor for all studied Au/Ni/AlGaN Schottky barrier diodes was from n = 1.3 to n = 1.6. The ideality factor reported in publication for Ni/AlGaN is within the range of 1.1–3 [20]. The vicinity of the ideality factor to unity in the current work indicates the good quality of the Schottky barrier.

Current–voltage characteristics for the graphene/AlGaN/GaN Schottky diode also demonstrated the exponential trend. However, one can see that contrary to the Au/Ni/AlGaN Schottky barrier diode, the characteristic of the graphene-based diode is not a straight line in a semi-logarithmic scale. 

This feature is known for graphene/semiconductor Schottky contact, and it can be attributed to the dependence of the Fermi level position in graphene on the bias and, as a result, to the dependence of the barrier height on the bias [21]. The characteristic for the graphene/AlGaN diode can be approximated by several exponents with different slope, as it is shown in Figure 5a. The ideality factors for different parts of the characteristics for this specific diode are 1.7, 1.8, 2.4. 

Equation (1) allows us to estimate the saturation current and the barrier height. However, the ideality factor n > 1 is an indication of the non-ideality of the barrier. Therefore, the barrier height for the diodes with the ideality factor significantly higher than unity should be considered only as an apparent barrier height. We found φ_b_ = 0.9 eV for the Au/Ni/AlGaN diode in Figure 5a, and within the range of φ_b_ = (0.7–1.1) eV for all studied diodes. The apparent barrier heights for the graphene/AlGaN diode in Figure 5a are 1.0, 1.15, and 1.2 eV. For all studied graphene-based diodes we found φ_b_ = (1.0–1.26) eV.

The barrier height for Ni/AlGaN Schottky barrier reported in the publications is within the range of (0.85–0.99) eV (see Reference [20] and references therein). The barrier height for graphene/GaN and graphene/AlGaN Schottky reported earlier is within the extremely wide range from 0.33 to 1.53 eV [13]. It was proved that apparent barrier height depends strongly on the defects concentration in the semiconductor and on Al mole fraction in AlGaN [13]. The maximum reported value of the graphene barrier height for AlGaN with the same as in the current study Al mole fraction is 1.25 eV [13]. The relatively high value of the apparent barrier height in this work is an indication of low defects concentration in AlGaN.

Figure 5b shows two examples of the representative graphene gate finFETs transfer current–voltage characteristics with a subthreshold slope of ~1.3 and ~8 orders of magnitude on/off ratio. It is interesting to note that, contrary to the forward current-voltage characteristic of the Schottky diode (Figure 5, the exponential (subthreshold), part of the transfer current–voltage characteristic is an exponent without any noticeable deviation. Although the barrier height should depend on the reverse bias as well, this dependence might be negligible in comparison with the total negative voltage V_g_ < −1.0 V.

**Figure 6:**
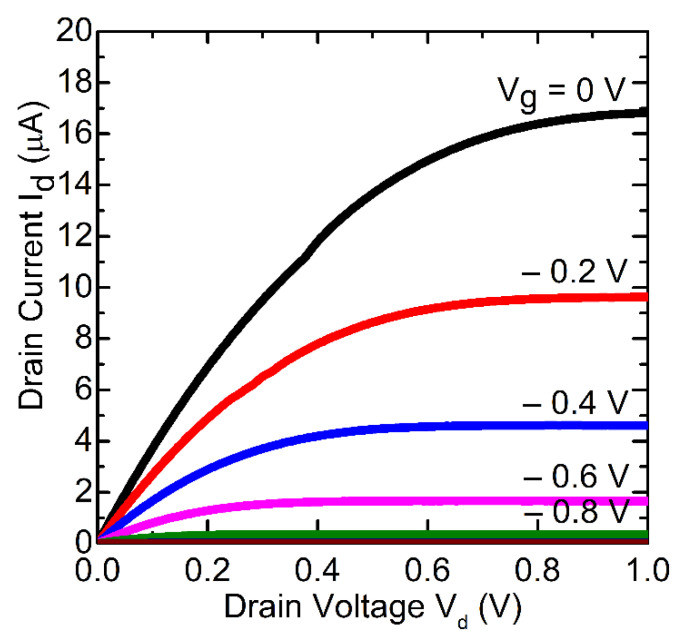
Output current-voltage characteristics of finFETs with graphene gate.

The subthreshold leakage current was below 0.1 pA, which is an indication of low gate leakage current. Several measurements of transfer characteristics during a 6 months period did not indicate any noticeable change in their shape. An example of the output current-voltage characteristics is shown in Figure 6.

The noise spectra of the finFETs drain current fluctuations had the form of 1/f a with a = 0.95–1.05. In the linear regime the spectral noise density of the drain current fluctuations, $S_{I}$, was always proportional to the current squared.

**Figure 7:**
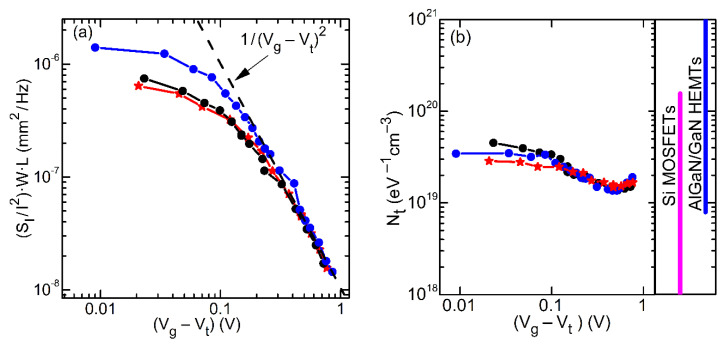
Dependences of noise on the gate voltage swing (V_g_ − V_t_) in the linear regime at f = 10 Hz for the representative devices; V_d_ = 0.1 V (**a**) and effective trap density Nt as a function of the gate voltage swing (V_g_ − V_t_) for AlGaN/GaN HEMTs with graphene gate (**b**).

The dependences of the relative spectral noise density $S_{I}/I^{2}$ on the gate voltage swing (V_g_ − V_t_) at frequency f = 10 Hz for three representative graphene gate transistors are shown in Figure 7a.

In the McWhorter model, the spectral noise density of the drain current fluctuations $S_{I}/I^{2}$ in the linear regime is given by [22]:

(2) $$\frac{S_{I}}{I^{2}}=\frac{k_{B}\cdot T\cdot N_{t}}{\gamma \cdot f\cdot W_{g}\cdot L_{g}\cdot n_{s}^{2}}$$

where f is the frequency, N_t_ is the effective trap density, $W_{g}\;\mathrm{and}\;L_{g}$ are the gate width and length, respectively, n_s_ is the concentration, and γ is the attenuation coefficient of the electron wave function under the barrier:

(3) $$\gamma =\frac{4\cdot \pi \cdot \sqrt{2\cdot m*\cdot \Phi }}{h}$$

Here m* is the electron effective mass, h is the Planck’s constant, and Φ is the tunneling barrier height at the interface. In Si MOSFETs, the γ value is usually taken to be γ = 10^8^ cm^−1^. For the AlGaN/GaN system, the attenuation coefficient is somewhat smaller: γ ≅ 0.45 × 10^8^ cm^−1^ [23].

Since in the linear regime n_s_ ~ (V_g_ − V_t_), the McWhorter model predicts the dependence of noise on the gate voltage as $S_{I}/I^{2}\;\sim \;{\left(V_{g}\;-\;V_{t}\right)}^{-2}$. In many publications on noise in AlGaN/GaN transistors, deviations from this law have been reported (see, for example, References [24,25]). These deviations from the McWhorter model are usually attributed to the influence of the contact resistance, gate leakage current, mobility fluctuations, and dependence of the trap density N_t_ on energy (gate voltage). As seen in Figure 7a, the gate voltage dependence of noise in studied devices is well described by the McWhorter model, unless the gate voltage swing is small, in which case the model is not applicable. Therefore, Equation (2) allows estimating the effective density of traps responsible for the noise.

The effective trap density can be also found from the input gate voltage noise

(4) $$S_{V_{g}}=\left(\frac{S_{I}}{I^{2}}\right)\cdot \left(\frac{I_{2}}{g_{m}^{2}}\right)=\frac{k\cdot T\cdot N_{t}\cdot q^{2}}{\gamma \cdot f\cdot W\cdot L_{g}\cdot C^{2}}$$

where g_m_ is the external transconductance, q is the electron charge, and C is the gate capacitance per unit area. This method allows us to get rid of the uncertainty of the threshold voltage and take into account the possible influence of the contact resistance [23].

Figure 7b compares the effective trap density as a function of the gate voltage swing extracted using Equation (4) for the same transistors as in Figure 7a. The right panel indicates the ranges of the trap densities published for Si MOSFETs with high-k dielectric and for similar AlGaN/GaN transistors with the metal Schottky gate [23,24,25,26,27,28,29,30,31,32].

One can see that the effective trap density for studied devices only weakly depends on the gate voltage, reflecting the $S_{V_{g}}$ dependence on V_g_. This effect can be due to the dependence of the trap density on the energy. At high gate voltage, the trap density is close to 10^19^ eV^−1^ cm^−3^. Similar or higher values of the effective trap density within the range of (10^18^–10^20^) eV^−1^ cm^−3^ were found in the majority of publications on Si MOSFETs with a high-k dielectric (see References [26,27,28,29,30,31]). We are aware of only one study of noise in high-k Si MOSFET that reported a very small trap density of N_t_ ≅ 10^17^ eV^−1^ cm^−3^ [31]. Other studies of noise in AlGaN/GaN HEMTs with metal gate indicated N_t_ = (10^18^–10^20^) eV^−1^ cm^−3^ (see Figure 7b and References [23,24,25,32]). We also found a similar range of effective trap density N_t_ = (2.3 × 10^19^–1.7 × 10^20^) eV^−1^ cm^−3^ in AlGaN/GaN HEMT structures with Ni/Au gate.

Although AlGaN/Schottky barrier interface is relatively far from the two-dimensional channel, it is known that this interface still may affect the noise originated from the channel. In Reference [32], it was shown that N_2_O or O_2_ plasma oxidation treatment of AlGaN surface strongly increases the noise. On the other hand, AlGaN/GaN transistors with insulated Al_2_O_3_ or SiO_2_ gate have shown to have lower noise [33]. Therefore, one can assume that graphene gate might make AlGaN surface vulnerable to air contamination and lead to the noise increase. However, our study showed similar noise levels and effective trap densities for graphene and Ni/Au Schottky gates finFETs.

## 4. Conclusions

In summary, we fabricated and studied graphene gate AlGaN/GaN finFETs. Study of the vertical transport in graphene/AlGaN Schottky diodes indicated the dependence of the Schottky barrier on the bias. The apparent barrier heights for the graphene/AlGaN diodes were found to be within the range of φ_b_ = (1.0–1.26) eV. The relatively high value of the apparent barrier height in comparison with other published results is an indication of low defect concentration in AlGaN and good quality of the interface. AlGaN/GaN finFETs demonstrated good characteristics with ~8 order of magnitude on/off ratio and subthreshold slope of ~1.3. The effective trap density found from the low-frequency noise measurements was ≥10^19^ eV^−1^ cm^−3^, which is of the same order of magnitude as in the transistors with Ni/Au Schottky gate. This is an indication that graphene forms a high-quality Schottky barrier to AlGaN which can serve as a gate for various types of transistors. It is important that the open surface of graphene does not deteriorate electrical and noise properties of 2D gas on the AlGaN/GaN interface. These results pave the way towards transparent high-temperature GaN-based electronics and to study Coulomb interactions in the graphene/AlGaN/GaN system.
